# Formation of the Long-Lived Parent Anion upon Electron Attachment to Menadione

**DOI:** 10.1021/acs.jpca.5c07629

**Published:** 2026-02-25

**Authors:** Farhad Izadi, Andrzej Pelc, João Ameixa, Fábris Kossoski, Stephan Denifl

**Affiliations:** a Institut für Ionenphysik und Angewandte Physik und Center for Molecular Biosciences Innsbruck, 27255 Universität Innsbruck, Technikerstraße 25, A-6020 Innsbruck, Austria; b Department of Biophysics, Mass Spectrometry Laboratory, Maria Curie-Skłodowska University, Pl. M. Curie-Skłodowskiej 1, 20-031 Lublin, Poland; c Institute of Chemistry, Hybrid Nanostructures, 26583 University of Potsdam, Karl-Liebknecht-Str. 24-25, 14476 Potsdam, Germany; d Centro de Física e Investigação Tecnológica (CEFITEC), Department of Physics, NOVA School of Science and Technology, NOVA University Lisbon, Caparica 2829-516, Portugal; e Laboratoire de Chimie et Physique Quantiques (UMR 5626), Université de Toulouse, CNRS, Toulouse 31062, France; f Department of Chemistry and Biochemistry, Texas State University, San Marcos, Texas 78666, United States

## Abstract

Menadione is a multifunctional molecule involved in critical biological processes such as blood coagulation, redox regulation, and cellular metabolism. Understanding its electron attachment properties and capacity to form stable anions is essential for elucidating its function in biological environments. In this study, we investigated electron attachment to menadione using a crossed electron-molecular beam experiment, complemented by quantum chemical and electron scattering calculations. Upon electron attachment, the efficient formation of the parent molecular anion is observed. Its signal extends from 0 to 2.5 eV, with pronounced peaks at ∼0 and 0.7 eV, assigned to the formation of different precursor anion states. Two fragment anions, namely, C_2_H_2_^–^ and CH_3_^–^, were also detected. In contrast to the parent anion, their formation occurs with significantly lower efficiency and only at higher electron energies, above 4 eV, consistent with the higher energy thresholds required for dissociative electron attachment. Our findings show, on the one hand, that the metastable parent anion of menadione has a relatively long lifetime, which may be further extended in biological environments due to solvent effects, and, on the other hand, that it is structurally stable in the interaction with low-energy electrons.

## Graphical abstract

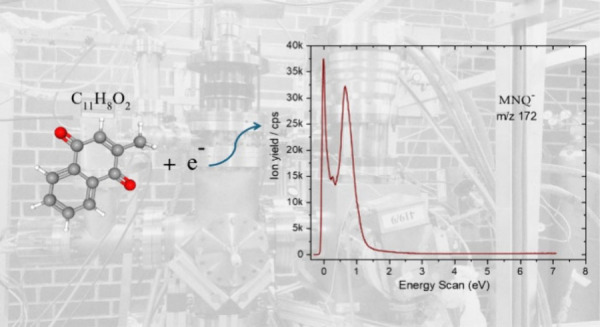

## Introduction

Menadione (2-methyl-1,4-naphthoquinone, C_10_H_5_O_2_CH_3_, MNQ), also known as vitamin K_3_, is a synthetic analogue of naturally occurring vitamin K. MNQ belongs to the family of quinones. This family is a diverse and biologically relevant group of compounds characterized by the presence of electron-withdrawing carbonyl groups conjugated within an aromatic ring system. This structural feature enables extensive delocalization of electrons throughout the π system, which is the basis for their unusual redox behavior. Quinones are capable of reversible redox reactions, acting as both electron acceptors and donors, thereby playing versatile roles in redox-mediated biochemical and chemical processes.^1^ Their redox flexibility allows them to participate in electron transfer across different redox couples, often accompanied by essential protonation steps.^2,3^ Typically, the reduction of a quinone proceeds via a one-electron transfer forming a semiquinone radical anion, which upon protonation leads to the fully reduced hydroquinone species.^1,4,5^ The one-electron reduction generates a highly reactive semiquinone radical, which then may be involved in the formation of reactive oxygen species (ROS) through oxygen interaction, leading to oxidative stress and cytotoxic effects.^6,7^ In contrast, the two-electron reduction followed by protonation contributes to the anti tumor action of quinone-based drugs via reductive arylation and DNA cross-linking.^8,9^ Cytotoxicity and therapeutic efficacy are closely tied to the electrochemical potential of the semiquinone intermediate, which is further modulated by the protein and enzyme environment affecting electron loss and quinone stabilization.^6,10^ The reduction of quinones in aprotic solvents, proceeds via two reversible or quasi-reversible one-electron transfer steps, yielding a radical anion and a dianion. These intermediates’ stability and reactivity are primarily governed by the electronic structure of the quinone, particularly by the energy of the lowest unoccupied molecular orbital (LUMO). Substituent effects via electron-donating or electron-withdrawing groups modulate these properties by changing LUMO energies, thereby influencing redox potentials and the nucleophilicity of reduced species. Additionally, non-covalent interactions such as hydrogen bonding and proton transfer also play a significant role in modulating redox behavior.^11,12^

The dual electron–proton behavior of quinones is not only fundamental to quinone chemistry but is also critical in many biological systems. Quinones serve as key cofactors in energy transduction, particularly within the electron transport chains of mitochondria, bacteria, and chloroplasts. They are integral to cellular respiration,^13^ photosynthesis,^14^ and redox cycling in soil ecosystems.^15^

MNQ was firstly identified for its role in blood coagulation–hepatic biosynthesis of blood clotting factors.^16^ Subsequent studies have revealed its broader biochemical significance, including redox cycling, cellular energy metabolism,^17^ and potential therapeutic applications.^18^ Similarly to the naturally occurring vitamins K_1_ (phylloquinone) and K_2_ (menaquinones), pure menadione is water insoluble and requires enzymatic conversion to its biologically active form, menaquinone-4 (MK-4). MK-4 has been shown to regulate gene expression related to bone formation and inflammation.^19^ Moreover, MK-4 mitigates oxidative damage in neuronal cells, which has relevance to neurodegenerative diseases (Alzheimer and Parkinson). MK-4 appears to enhance mitochondrial function, reduce lipid peroxidation, and modulate apoptotic pathways, all of which contribute to its protective effects.^20^ These properties have increased interest in vitamin K analogues as potential drugs in therapies of neurodegenerative diseases, highlighting the broad physiological significance of MNQ metabolism.^20^

MNQ has also been investigated for its anticancer properties, primarily due to its ability to induce oxidative stress in cancer cells. In vitro studies have shown that MNQ can selectively destroy tumor cells by generating high levels of ROS, disrupting mitochondrial function, and depleting cellular antioxidants like glutathione.^21–23^ However, its therapeutic application is complicated by its narrow therapeutic window, as excessive ROS production can also harm healthy tissues. Excessive intake can lead to hemolytic anemia, liver damage, and kidney toxicity, primarily connected to uncontrolled ROS generation and depletion of cellular antioxidants.^24,25^ Due to its toxicity, current applications in human medicine remain limited, with preference given to safer vitamin K analogues like phylloquinone and menaquinones. However, it remains widely employed in animal nutrition. In agriculture and veterinary medicine, MNQ is widely used as a dietary supplement.

In the pharmaceutical industry, MNQ has been explored for its potential in drug development, particularly in combination therapies targeting oxidative stress-related conditions (e.g., with vitamin C).^26,27^ Some studies have investigated its use as a chemo- and radiosensitizer in cancer treatment, where it enhances the effects of both chemo- and radiotherapy.^28,29^

From a cancer therapy perspective, an effective sensitizer should preferentially target cancer cells while minimizing effects on healthy cells. Especially beneficial in this light are substances which may serve as sensitizers^30^ and additionally are essential for the proper functioning of the body, such as vitamins.^31,32^ Three primary mechanisms have been proposed to explain the activity of MNQ in cancer therapy: (i) the generation of ROS through quinone redox cycling and type II photosensitization (via energy transfer); (ii) covalent conjugation of MNQ with protein thiol groups, resulting in glutathione depletion and (iii) altered intracellular calcium levels; and the direct activation of transcription factors and other proteins via arylation by MNQ.^28,33^ In the first mechanism, MNQ undergoes redox cycling, accepting and donating electrons to produce superoxide and other ROS. There should be added that the role of oxygen in radiotherapy is critical: radiation induces DNA damage through the formation of free radical intermediates. Molecular oxygen, due to its high electronegativity, stabilizes these radicals by forming peroxides, leading to irreversible DNA damage. In hypoxic conditions, however, these radicals can be neutralized through proton transfer from cellular components, reversing the DNA damage and reducing treatment efficacy.^34^ In the second mechanism, MNQ forms covalent adducts, e.g., with cysteine residues on proteins, decreasing intracellular glutathione. The third involves direct electrophilic attack on nucleophilic residues of transcription factors, leading to their activation.

MNQ is also a molecule where one hydrogen atom in the naphthoquinone is replaced by the methyl group. The electronic properties of substituents on the quinone core can influence both the redox behavior of the electroactive moiety and the acidity of proton-donating or -accepting groups within the molecule, hence a MNQ molecule may serve as a model for other substituted naphthoquinones.^11^

Consequently, based on the above listed applications and properties of MNQ (and the whole quinones family as well), detailed investigation of electron transfer properties, protonation, and chemical reactivity is necessary for understanding redox processes. Special attention should be given to the formation of ionic intermediates, particularly the generation of negative ions from quinones during electron transfer reactions and interaction with electrons. In the latter case, electrons with low energies (<15 eV) are the most important. Moreover, the low-energy electrons play a central role in radiation chemistry, influencing physical and chemical processes initiated by ionizing radiation. The high-energy radiation used in cancer therapy can generate a large number of low-energy secondary electrons within cells. The energy distribution of secondary electrons from water radiolysis typically exhibits a most probable energy of about 9 eV, ranging from about 1 to 100 eV, depending mostly on the primary ion energy.^35^ These electrons may interact with biomolecules and therapeutic agents such as MNQ, potentially inducing further molecular damage. Therefore, understanding the fragmentation pathways and energetics of MNQ and other sensitizers upon low-energy electron interaction is essential for optimizing therapeutic strategies, including radiation energy and dosage parameters.

Despite the great importance of the properties of MNQ anions, to the best of our knowledge no studies of electron capture by this compound have been conducted so far. However, it is worth mentioning the comprehensive study of Bull et al. on the anions of MNQ as probed by photoelectron spectroscopy.^36^ A little more information can be found on electron attachment to MNQ precursors such as naphthalene and naphthoquinone and their derivatives.^11,37–45^ The situation is not much better for studies of positive ions formed from MNQ. The only mass spectrum for positive ions can be found in the NIST database.^46^ The electron impact (EI) mass spectrum of MNQ, as reported in the NIST database, displays multiple ion groups around the *m*/*z* (mass to charge ratio) of 18, 28, 39, 50, 63, 76, 89, 104, 115, 129, 144, 157 and 172. The most prominent peaks are observed at *m*/*z* of 172, corresponding to the parent cation, as well as at *m*/*z* of 115, 104 and 76, which may be assigned to C_9_H_7_^+^, C_8_H_8_^+^, and C_6_H_4_^+^, respectively.

In recent years, electron attachment processes to various quinones were systematically investigated at the Innsbruck Laboratory. For coenzyme Q_0_ (CoQ_0_), we demonstrated a stabilization mechanism through an initially formed dipole-bound state, confirming CoQ_0_ as a model for electron withdrawing behavior in the ubiquinone family, in contrast with larger ubiquinones, where anion stability occurs through higher energy resonances.^47^ Also, we studied dissociative electron attachment (DEA) to CoQ_0_ and its reduced analogue CoQ_0_H_2_, identifying pathways leading to distinct anionic fragments.^48^ Finally, for methyl-*p*-benzoquinone (MpBQ), we observed the efficient formation of the parent anion at higher energies, in close parallel with the case of *p*-benzoquinone (pBQ).^49^ In comparison with the latter, we also found that the methyl group in MpBQ introduces new DEA reactions while quenching others.

The above-mentioned considerations motivated us to conduct a mass spectrometric study of the low-energy electron interactions with the MNQ molecule. Experimental results are supported by theoretical calculations to elucidate both the fragmentation pathways responsible for the formation of the observed anionic species and the character of the bound and resonant anion states.

## Experimental Method

The electron attachment spectrometer employed in this study consists of a molecular beam source, a high-resolution hemispherical electron monochromator (HEM), and a quadrupole mass spectrometer (QMS), equipped with a pulse-counting system for analyzing ionic products. A detailed description of the apparatus is available in a previous publication.^50^

The investigated compound, MNQ, is a solid at room temperature, with a low vapor pressure of 0.025 Pa at normal conditions, and a melting point of 380 K.^51^ For this reason, the MNQ sample was heated gradually in the resistively heated oven to attain a temperature of 329 K, at which we do not observe thermal decomposition and the ion signal is relatively high. The thermal stability of the sample was checked by the measurement of positive ion mass spectrum at several sample temperatures. The vapor was directly introduced into the HEM interaction region via a copper capillary. The vapor flow into the interaction region was controlled by monitoring the pressure in the main vacuum chamber, which houses both the HEM and the QMS. Throughout the measurements, the pressure was kept at approximately 2 × 10^–5^ Pa, ensuring single collision conditions.

Anions produced by low-energy electron attachment were extracted using a weak electrostatic field (∼0.6 V/cm) and directed into the QMS for mass analysis (mass resolution *m*/Δ*m* ≈ 120 at *m*/*z* 122, where Δ*m* refers to the full width at half maximum, FWHM, of the mass peak). The ions were detected by a channeltron electron multiplier. Residual electrons were collected by a Faraday cup, and the electron current was monitored using a picoammeter.

To calibrate the electron energy scale and determine the energy spread of the HEM, the well-characterized DEA process of Cl^–^ formation from CCl_4_ was employed. This reaction exhibits two resonances at 0 and 0.8 eV.^52,53^ The 0 eV peak was used for energy calibration and energy spread determination (FWHM), while the 0.8 eV resonance, with a known cross section (CS) of 5 × 10^–20^ m^2^,^53^ served as a reference to estimate the CS of electron attachment to MNQ.

Estimated values for associative attachment CS were obtained by comparing the anion signal intensities from MNQ to those from CCl_4_ under identical spectrometer conditions. Differences in molecular density between MNQ (introduced via capillary) and CCl_4_ (introduced as stagnant gas) were also considered. However, this method provides only an approximate CS estimation, as done in earlier studies of DEA.^54–56^ Systematic uncertainties such as ion discrimination in the HEM region, variation in ion transmission efficiency in the QMS, and different ion detection efficiencies in the channeltron were not corrected for. Consequently, the estimated CS values may deviate by up to an order of magnitude from the absolute values.

Resonance energies were determined by fitting Gaussian functions to the experimental ion yield data. Anion appearance energies (AE) were estimated using the method described by Meißner et al., with the AE calculated as AE = EG_max_ – 2σ, where EG_max_ is the energy of the Gaussian peak maximum and σ is its standard deviation.^57^ The fitting was performed using the Origin software.

In the present measurements, the electron beam had an energy resolution (FWHM) of 120 meV and a current of 30 nA. This resolution was selected as a compromise between maximizing ion signal and maintaining sufficient energy discrimination to resolve resonances. The estimated uncertainty in the presently reported peak energies and AEs is ±0.1 eV. The HEM was continuously heated to 360 K to prevent surface charging. MNQ (98% purity) was obtained from Merck (Vienna, Austria).

## Theoretical Methods

For better insight into the electron attachment processes, we performed electron scattering calculations using the Schwinger Multichannel (SMC) method.^58^ Only the computational details relevant to this work are provided below; the full methodology is described in detail elsewhere.^59^ The scattering calculations were carried out at the optimized geometry of the neutral ground state of MNQ, obtained using the CAM-B3LYP functional with Dunning’s aug-cc-pVDZ basis set, as implemented in *Gaussian 16*.^60^ Only the elastic scattering channel was considered in our model. The electronic structure of the target was described at the restricted Hartree–Fock level of theory. For carbon and oxygen atoms, a basis set of 5s5p2d Gaussian functions was used, whereas for hydrogen atoms, 3s Gaussian functions were employed. The Gaussian exponents can be found in ref 61.

The scattering wave function was constructed as a linear combination of configuration state functions (CSFs), which are antisymmetrized products of a target electronic state and a scattering orbital representing the continuum electron. The scattering orbitals were expressed as modified virtual orbitals (MVOs), obtained by diagonalizing a modified Fock operator with an effective charge of +6. Two types of CSFs were included in the calculations. The first type consisted of the Hartree–Fock ground-state wave function combined with all MVOs. The second type involved a subset of single excitations of the target (including both spin multiplicities) coupled with MVOs, selected based on an energy cutoff criterion involving differences in orbital energies, as described in ref 62. In this study, an energy cutoff of 1.6 Hartree was applied, yielding a total of 21829 CSFs. No vectors were removed based on the smallest singular values of the SMC denominator matrix. Due to the high computational cost of the scattering calculations, only the A″ symmetry was considered, as the present low-energy resonances are found in this symmetry. Resonance energies and widths were extracted by fitting the computed CS to a Lorentzian function superimposed on a second-order polynomial background. Resonance assignments were based on the eigenvectors of the Hamiltonian matrix in the CSF space whose eigenvalues are close in energy to the features observed in the CS. The orbital representation of the shape resonances was obtained by projecting the CSFs of the first type onto the Hartree–Fock target state.^63^

Although fixed-nuclei SMC calculations yield electron attachment energies, they do not account for vibrational relaxation. To explore possible dissociation pathways, we computed thermochemical thresholds and electron affinities (EAs) using the G4MP2 method,^64^ as implemented in *Gaussian 16*.^60^ The estimated uncertainty for these thermochemical values is approximately ±0.1 eV.^64,65^

## Results and Discussion

In the present study on electron attachment to the MNQ molecule, only three distinct anionic fragments were observed, with *m*/*z* ratios of 172 (MNQ^–^), 26 (C_2_H_2_^–^), and 15 (CH_3_^–^). In Figure 1, the anion efficiency curves for all the negatively charged species in the electron energy range of about 0–10 eV are presented. The ion signal intensities are reported in relative units, although the yields of different anions share the same scale. The formation of anions occurs within the whole electron energy range investigated here. In the lower energy range (below 1 eV), only the parent anion is formed, while the two other anions are only observed at higher energies. Surprisingly, we did not observe the (M – H)^−^ anion, even though this is a commonly observed species in DEA to organic molecules.^66,67^ In particular, (M – H)^−^ ions have been observed in studies of the interaction of low-energy electrons with several quinones.^40,49,68^ The corresponding peak positions and AEs obtained in the present experiment are summarized in Table 1, together with the calculated thermochemical thresholds and both the calculated and previous experimental EAs of the neutral fragments.^46^

**Figure 1:**
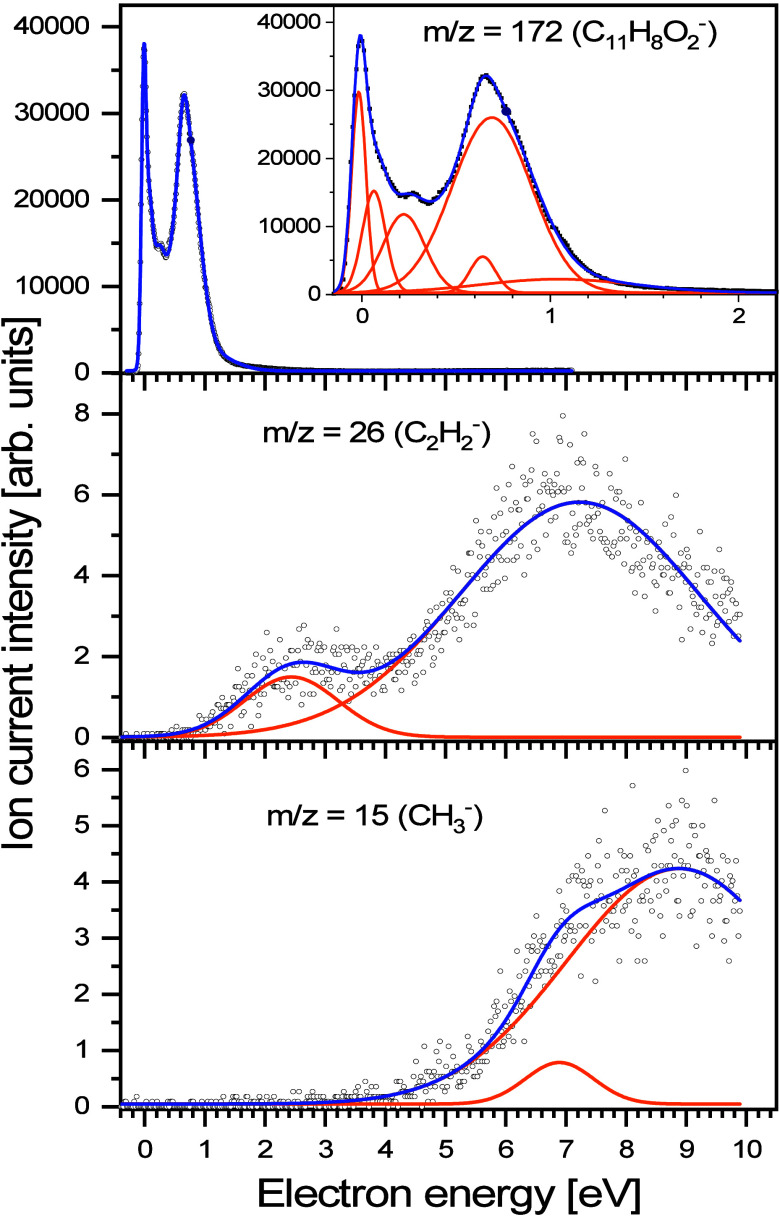
Anion efficiency curves of the anions observed upon electron attachment to MNQ. Gaussian peaks fitted to the experimental data, which were used to estimate the appearance energy and the peak position, are represented by orange lines. For a better illustration of resonances in the parent ion, an ion yield magnification for the range of 0 to 2 eV has been added.

**Table 1:** Peak Positions with the Corresponding Appearance Energy in Parentheses, as Observed in the Ion Yield Formed upon Electron Attachment to MNQ, Presently Calculated Thermochemical Thresholds, and Calculated and Experimental Electron Affinities for the Neutral Structures of the Measured Anions

				Electron affinity of the neutral structure (eV)	
*m*/*z*	Structure	Peak energy (appearance energy) (eV)	Calculated thermochemical threshold at 298 K (eV)	Theory	Experiment^46^
172	C_11_H_8_O_2_^–^	0, 0.06, 0.22, 0.64 (0.5), 0.69 (0.27), 1.04 (0.24)	–1.96	1.95	1.77
26	C_2_H_2_^–^	2.43 (0.89), 7.22 (3.24)	3.38	0.53	0.48
15	CH_3_^–^	6.89 (5.75), 8.88 (5.14)	4.35	0.04	0.13

Figure 2 presents both the experimentally measured total electron attachment CS and the theoretically predicted elastic CS for the A″ symmetry component. The experimental data is virtually identical to the contribution from the parent anion but also accounts for the minor contributions from the two DEA channels. Peaks in the calculated elastic CS correspond to resonant anion states, which arise from temporary capture of the incident electron by the molecule. While the calculated and experimental CSs are not directly comparable in a quantitative sense, the theoretical elastic CS provide insight into the resonant states that initiate the different electron attachment channels. Between 0 and 1 eV, we identify a clear correspondence between the two main experimental features and the two peaks obtained in the calculations. It is important to note that the calculated resonances appear narrower than the experimental ones, which is a known limitation of the fixed nuclei approximation.^69,70^ Another observation is that the peaks in the presently calculated CS are shifted toward higher energies (by around 0.2 eV) relative to the peaks in the experimentally determined electron attachment CS, which likely reflect inaccuracies in the theoretical model. The calculated peak value of the elastic CS is approximately 220 × 10^–20^ m^2^, whereas the experimental electron attachment CS reaches about 1.9 × 10^–20^ m^2^, roughly two orders of magnitude lower. Bearing in mind the fixed-nuclei approximation of the calculation and the large uncertainty in the experimental estimate, the large difference in CSs suggests that electron autodetachment is the prevailing decay channel. The experimental and theoretical CSs determined for MNQ are significantly higher than the CS value estimated by Christhophorou and Blaunstein^43^ for naphthalene (C_10_H_8_), with its two aromatic rings structurally similar to MNQ, reported to be less than 1.27 × 10^–23^ m^2^. It can also be noted that such large CS only appear at low energies, between 0 and 1 eV. By going beyond the fixed-nuclei approximation, one could in principle compute Frank–Condon factors between the neutral ground state and a particular anionic state. The single and narrow peak from our calculation would be replaced by a broader band containing a series of vibronic transitions (modulated by Frank–Condon factors), and having a smaller maximum cross section. Even though this would bring the maximum calculated cross section closer to the experimental one, the very large difference in CSs can still be attributed to the autodetachment channel. The temperature, which defines the distribution of initial vibrational levels, could have some impact on the attachment cross section, but less so on the elastic cross section, and therefore is not expected to play a major role in the present comparison between experiment and calculations.

**Figure 2:**
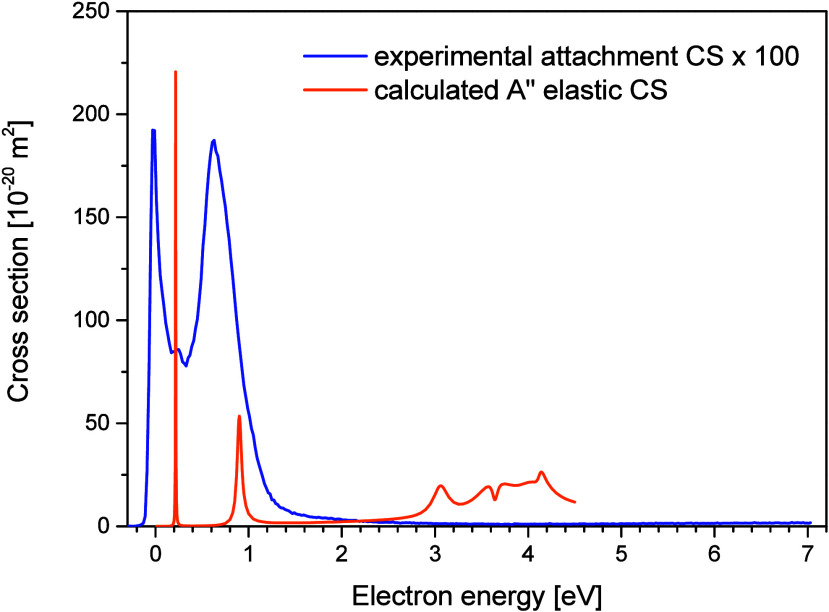
The total experimental electron attachment cross section from MNQ (blue line) and theoretically obtained elastic cross section for the A″ symmetry (orange line).

Table 2 summarizes the resonance energies determined both theoretically and experimentally, along with the corresponding assignments of the anionic states. For comparative analysis, analogous data for naphthoquinone (which differs from MNQ by the absence of the methyl group) have been included based on previously published theoretical and experimental studies.^39,45^ The molecular orbitals relevant to the resonances of MNQ are illustrated in Figure 3, being similar to those of naphthoquinone (not shown). Despite the close parallel in character, the resonance energies appear systematically higher in MNQ, with larger differences for the higher lying resonances. The same effect has been previously noted for the analogous cases of MpBQ and pBQ.^49^ The larger gaps for higher lying resonances are probably artifacts resulting from the comparison of different theoretical models: an empirical scaling relation in the case of naphthoquinone,^39^ and a particular ansatz for the scattering wave function in our SMC calculations for MNQ. One or both models may favour the description of some of the resonances, producing artificially larger energy gaps between analogous resonances. Indeed, by employing the same scaling relation of ref 39 to MNQ, we do not find clear trends as a function of energy. Still, the resonances do appear higher in energy in MNQ than in naphthoquinone, probably a real effect induced by the methyl group.

**Figure 3:**
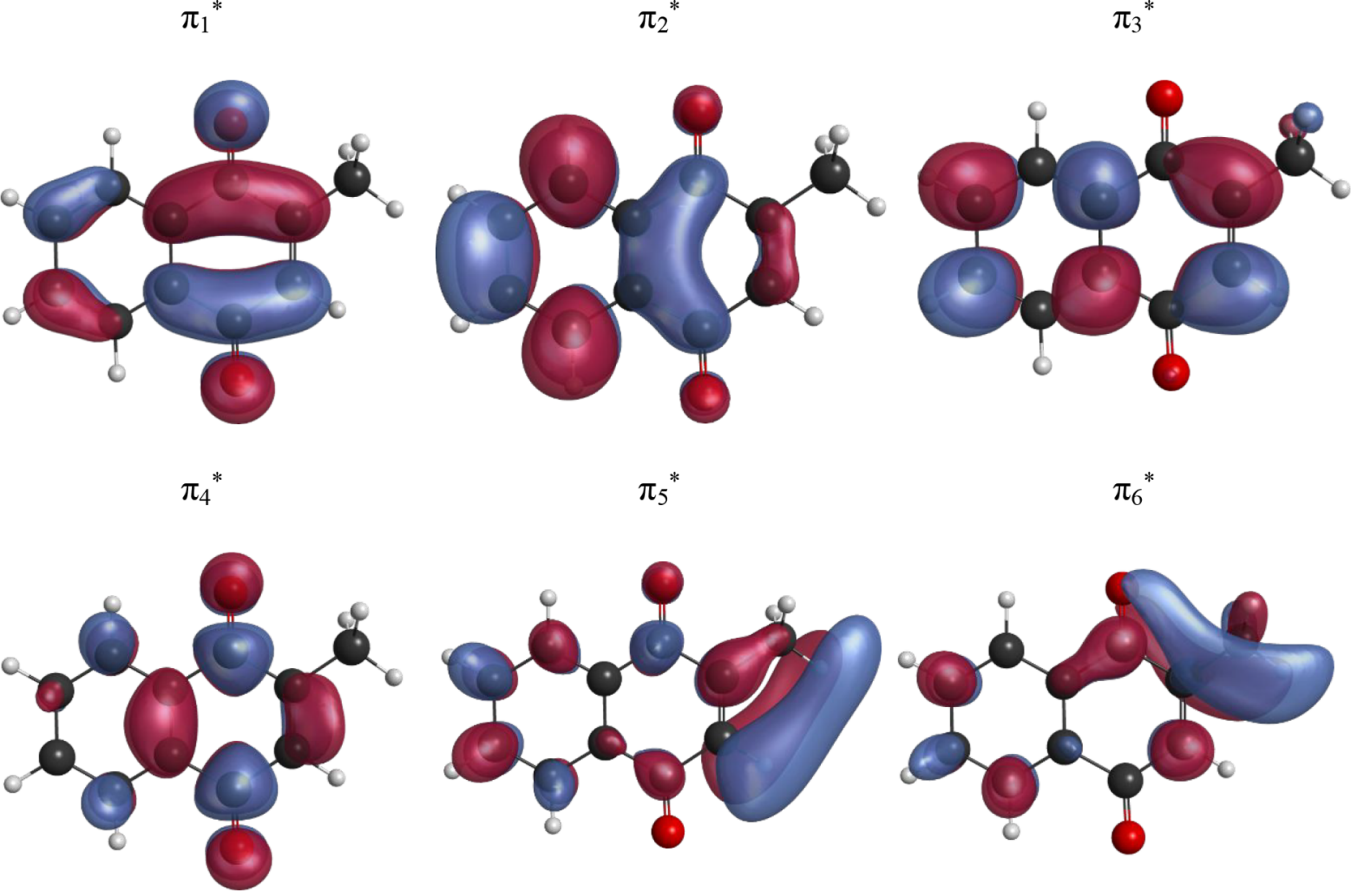
Virtual (π*-type) molecular orbitals relevant to the low-energy resonances of MNQ.

**Table 2:** Theoretical Resonance Energies (with Corresponding Widths in Parentheses), Given in eV, for the Anionic States of MNQ, Labeled by Their Dominant Electronic Configuration^a^

MNQ (present study)		1,4-naphthoquinone^39^	
Anion state	Theory	Theory	Experiment
π_1_*	–1.54	–1.64	–1.81
π_2_*	0.217 (0.002)	–0.04	0.2
π_3_*	0.901 (0.029)	0.37	0.59
π_4_*	3.067 (0.098)	1.80	1.75
π_5_*, π_6_*, core-excited resonances	3.55, 3.75, 4.14	2.70	2.41

a For comparison, the corresponding data for the analogous anionic states of naphthoquinone are also provided.

The lowest energy peak in the calculated elastic CS appears at 0.22 eV and corresponds to a shape resonance arising from electron capture into the π_2_* molecular orbital. The extra electron is located mostly at the benzene ring, with a smaller contribution from the quinone ring. The subsequent two peaks in the elastic CS, located at 0.90 eV and 3.07 eV, are attributed to π_3_* and π_4_* shape resonances. The former has contributions from the two rings, whereas the latter is mostly centered at the quinone ring. There are additional peaks at 3.55 and 3.75 eV, separated by a dip, and followed by a weak shoulder at 4.0 eV and another peak at 4.14 eV. These features have large contributions from the π_5_* and π_6_* one-particle configurations and from core-excited configurations involving the π_1_* and π_3_* orbitals. Due to the strong mixing of configurations and the proximity in energy, it is not possible to confidently assign the origin of each structure in the calculated CS. MNQ also supports a valence bound anion, found at –1.54 eV in the SMC calculations, where the π_1_* orbital (localized mostly at the pBQ moiety) is occupied. In contrast, it does not have a strong enough dipole moment to support a dipole-bound anion. MNQ is also not expected to have low-lying σ* resonances which are more relevant in molecules containing heavier elements like for example chlorine and bromine^71^ as well as formic acid.^72^ While MNQ should have higher lying σ* resonances arising from the network of C–C/C=O bonds, they would appear considerably higher in energy than the π* resonances. Similarly, σ* resonances associated with C-H bonds typically appear at higher energies and are too short-lived, thus not being expected to play a significant role in the production of stable anions.

The following subsections present an analysis of the formation mechanisms of all anionic species generated via electron attachment to MNQ.

### C_11_H_8_O_2_^–^ (Parent Anion, MNQ^–^)

In the present study, the metastable parent anion of MNQ was detected. This anion is the most efficiently formed species upon electron attachment to MNQ, exhibiting an ion yield approximately 5000 times higher than that of the other anions formed via DEA. Notably, neither benzene nor naphthalene, which can be seen as structural precursors of MNQ, are capable to form parent anions with lifetimes sufficient for detection by mass spectrometry.^43,44,73^ Heinis et al. investigated the impact of substitution on the electronic properties of naphthalene and anthracene.^74^ They observed that introducing strongly electron-withdrawing groups such as CHO, CN, and NO_2_ significantly increases the EA of both molecules. By analogy, the carbonyl group (CO) should have a similar effect on EA due to its electron-withdrawing nature. Moreover the incorporation of two carbonyl groups, characteristic of quinones, into these aromatic systems will increase EA even more which then leads to stabilization of the parent anion, a feature commonly observed across the quinone family^39,49^ . Indeed, the parent anion has been observed in pBQ, MpBQ, and naphthoquinone, although with important differences in energy, as discussed below. It was also showed by Aguilar-Martinez et al. that reactivity and stability of naphthoquinones are significantly influenced by structural features such as substituents and intramolecular and intermolecular hydrogen bonding.^11^ In naphthoquinones, intramolecular hydrogen bonding stabilizes both the radical anion and dianion forms, shifting their redox potentials to less negative values. The electron-donating methyl groups increase the electron density within the quinone system, resulting in enhanced acidity and facilitating the reduction process of the quinone and its intermediate redox products.^75^

The EA of a neutral molecule plays a critical role in determining the stability of the resulting molecular anion, particularly with respect to electron autodetachment, which directly correlates with the anion’s lifetime. Benzene and naphthalene exhibit negative EAs.^46,74^ Such negative values result in short-lived parent anions due to rapid electron autodetachment. In contrast, our G4MP2 calculations for MNQ yield an EA of 1.95 eV. Our value is in reasonable agreement with earlier calculations (1.67 eV) and with the most recent experimental value of 1.63(6) eV.^36^

Analysis of the MNQ^–^ anion yield as a function of electron energy (Figure 1) reveals a structured feature at low energies, ranging from 0 to 2.5 eV. At first glance, three prominent peaks appear, at ∼0, 0.22, and 0.69 eV. However, they exhibit asymmetric profiles with noticeable high-energy tails, suggesting the presence of additional overlapping features. By fitting Gaussian functions to the experimental data, three additional and weaker features were identified, at 0.06, 0.64, and 1.04 eV. Before discussing the origin of these features, we will make a comparison with previous observations for related molecules. The presence of resonances above ∼0 eV threshold leading to the formation of stable molecular anions is a distinctive property of the quinone family^39,40,49^ highlighting their unique ability to stabilize excess electronic energy without undergoing rapid autodetachment or fragmentation. For instance, studies by Asfandiarov^39^ demonstrated that electron attachment to naphthoquinone leads to the formation of a parent anion via two prominent resonant channels, one at approximately 0 eV and the other around 0.9 eV, while the total ion signal extends across a broader range, up to 2 eV, indicating other possible resonances. This profile is very similar to what we observe for MNQ, showing that the methyl group plays a minor role in the stabilization of the anion. Indeed, the same general profile has been observed for a series of substituted naphthoquinones, although a third and distinct peak becomes visible in some.^40^ Similarly, investigations on anthraquinone have revealed the existence of three distinct resonances associated with parent anion formation, located at 0, 0.6, and 1.9 eV.^40^ In contrast, the parent anions of pBQ and MpBQ are observed at a single distinct peak, at 1.4 eV and 1.6 eV, respectively.^49^ These findings show that, starting with pBQ, the progressive extension of the conjugated π-system through the addition of one aromatic ring in naphthoquinone and two in anthraquinone, facilitates the activation of electron attachment channels. This reflects the increasing number of anionic resonances that appear upon conjugation of the π-system.

The prominent feature at 0.69 eV is likely due to formation of the π_3_* shape resonance, found at 0.90 eV in our scattering calculations. The origin of the peak at ∼0 eV is more puzzling. The analogue ∼0 eV peak in naphthoquinone and a series of derivatives has been proposed to originate from a vibrational Feshbach resonance of the π_1_* bound anion. While this is also a plausible mechanism for MNQ, we suggest direct formation of the π_2_* shape resonance (obtained at 0.22 eV in the scattering calculations) as an alternative mechanism. We notice that the equivalent π_2_* resonance in naphthoquinone was also found close to 0 eV.^39^ Interestingly, the parent anions of pBQ and MpBQ are not observed around 0 eV, even though their π_1_* bound anion is very similar in character to those of MNQ and naphthoquinone. This observation seems to favour our proposed stabilization mechanism through the π_2_* resonance. The presence of a distinguishable peak at 0.22 eV suggests that both mechanisms could play a role, although it could also be due to vibrationally excited levels. In any case, the prevailing channel enables the efficient formation of parent anions even at thermal electron energies, underscoring the unique electronic structure and stabilization mechanisms of quinone-based systems. We further notice that both mechanisms for the stabilization of the parent anion of MNQ differ from the one proposed for CoQ_0,_, which would involve the formation of a dipole-bound state.^47^ In the vibrational Feshbach resonance mechanism, the electron is captured into the LUMO and the excess energy is directly deposited into the vibrational degrees of freedom.^39^ In contrast, when the electron is captured into a higher lying molecular orbital, the initially formed excited anion may undergo internal conversion to the electronic ground state through one more conical intersections. If this process occurs in an ultrafast time scale, it may outcompete the electron autodetachment channel, resulting in the stabilization of the parent anion. This mechanism has been extensively investigated for the cases of pBQ^76^ and MNQ.^36^ These studies confirmed that the ground state anion can be efficiently recovered after it is photoexcited into shape resonances.

The exact position and width of the weak 0.64 eV feature is fit-dependent, hence this peak may be of limited physical significance, with no compelling evidence for a distinct anion state at that energy. It may be the interpreted as part of the 0.69 eV resonance and associated with specific excitation of vibrational modes. Finally, the weak and broad feature centered at 1.04 eV probably bears no special meaning and is simply needed to introduce some degree of asymmetry for the main 0.69 eV feature.

### *m*/*z* = 26 and 15 (C_2_H_2_^–^ and CH_3_^–^)

The other two anionic species detected in our study are formed much less efficiently than the parent anion (see Figure 1). Among these, the C_2_H_2_^–^ fragment requires significant molecular reorganization, as its formation involves the cleavage of at least two bonds within the aromatic rings of the MNQ structure. Interestingly, the corresponding positive ion has been previously identified in the EI mass spectrum of MNQ, suggesting a common fragmentation pathway.^46^ The formation of C_2_H_2_^–^ has also been observed in structurally related systems: benzene,^73^ 2,3-dimethoxy-5-methylhydroquinone (CoQ_0_H_2_),^48^ and MpBQ.^49^ In contrast, it has not been observed in DEA to pBQ. In the case of benzene, C_2_H_2_^–^ formation was reported across two broad resonance regions (2 to 6 eV and 6 to 12 eV), with a prominent maximum around 9 eV. Notably, this fragment is characterized by the strongest ion signal among all DEA products for benzene. For CoQ_0_H_2_, C_2_H_2_^–^ formation occurs via an asymmetric peak at 2.0 and another at 5.9 eV, superimposed on a non-resonant ion signal attributed to ion-pair formation. In MpBQ, features were observed at 8.1 and 9.7 eV. In the present study, the formation of the C_2_H_2_^–^ anion was found to occur via two distinct resonance peaks, located at 2.43 and 7.22 eV. The corresponding AEs were determined to be 0.89 and 3.24 eV, respectively. The present thermochemical calculations indicate energy thresholds of 3.38 eV when the neutral counterpart is coumarin or isocoumarin, 3.40 eV when 1,3-indandione is produced, and 3.80 eV in the case of chromone. Higher energy isomers also exist. While we cannot conclude which neutral fragments are produced, 1,3-indandione seems likely, having practically the lowest thermodynamical threshold and requiring little molecular rearrangement after elimination of the C_2_H_2_^–^ anion from MNQ. Considering the estimated uncertainties in both the theoretical calculations and experimental AE values (each approximately ±0.1 eV), the higher energy structure (centered at 7.22 eV and starting at 3.24 eV) is consistent with formation of 1,3-indandione or (iso)­coumarin as the neutral counterpart of C_2_H_2_^–^. In contrast, the low-energy feature at 2.43 eV is most likely attributed to impurities in the MNQ sample, potentially due to residual benzene. An alternative explanation for the low-energy resonance could involve electron attachment to vibrationally excited MNQ molecules. However, given the relatively low temperature of the sample under the experimental conditions, this explanation appears unlikely.

Overall, the occurrence of high-energy resonances associated with C_2_H_2_^–^ formation across related aromatic systems (benzene, CoQ_0_H_2_, MpBQ and MNQ) supports the hypothesis of shared DEA channels for this molecular class. A notable exception is pBQ, where the C_2_H_2_^–^ fragment is not observed among the various other DEA channels.^77^

A possible explanation for the absence/very low abundance of a C_9_H_6_O_2_^–^ signal (corresponding to the loss of neutral C_2_H_2_) may be the rapid autodetachment of the extra electron or more likely, the presence of efficient fragmentation pathways that makes impossible stabilization of the intact fragment anion. In fact, we found a weak ion yield at *m*/*z* 145 (C_9_H_6_O_2_ – H)^−^ in the mass spectrum at the electron energy of 8 eV (close to the resonance leading to C_2_H_2_^–^). However, this signal was approximately five times weaker than that at *m*/*z* 26, and due to the expectable poor statistics, we did not measure its energy profile.

The weakest anion signal in our study corresponds to the formation of the CH_3_^–^ anion, comparable in intensity to that of C_2_H_2_^–^. It displays a very weak signal at 6.89 eV and a broader feature centered at 8.88 eV. This process likely involves the cleavage of a single C–C bond, resulting in the elimination of a negatively charged methyl group. Notably, this DEA channel was not observed in MpBQ, despite its smaller size. This suggests that other minor DEA reactions, seen in MpBQ but not in MNQ, would quench production of CH_3_^–^ in the former. Interestingly, CH_3_^–^ was not observed in DEA to related but larger species, like methylated anthraquinones, where (M – CH_3_)^−^ was detected instead.^40^ It is worth mentioning that Ameixa et al. also observed formation of CH_3_^–^ for CoQ_0_ (at 8.1 and 9.7 eV) and for CoQ_0_H_2_ (at 9.0 eV),^48^ which however have more methyl groups than MpBQ.

The measured anion yield for CH_3_^–^ in the case of MNQ spans a broad electron energy range, extending from approximately 4 eV to beyond 10 eV. Upon detailed analysis, two resonance peaks were identified at 6.89 and 8.88 eV, with corresponding AEs of 5.75 and 5.14 eV, respectively. The lowest calculated thermochemical threshold for the DEA channel resulting in CH_3_^–^ and its neutral counterpart is 4.35 eV, approximately 0.8 eV lower and thus consistent with the lowest AE determined experimentally.

The absence of (M – CH_3_)^−^ is somewhat puzzling, considering that its production has a smaller reaction threshold (2.31 eV) than the one for CH_3_^–^ (4.35 eV), which we have observed. However, because of the lower threshold, the (M – CH_3_)^−^ species would have more excess energy, favouring further decay, potentially into C_2_H_2_^–^ and a neutral counterpart. The observation that the yields of C_2_H_2_^–^ and CH_3_^–^ have similar profiles also suggests that they may be formed from the same temporary negative ion state(s).

## Conclusion

Menadione plays multifunctional roles in biological systems, notably in blood coagulation, redox homeostasis, and mitochondrial metabolism. Due to its redox-active quinone structure, MNQ has also been proposed as a potential radiosensitizer and chemotherapeutic adjuvant. Here, we investigated the mechanisms of low-energy electron attachment to MNQ using a combined experimental and theoretical approach, with the goal of elucidating its capacity to act as an electron acceptor. Though the present results are limited to gas-phase measurements, they establish a clear baseline for MNQ anion formation and stability that is necessary for any further interpretation of its behavior in more complex chemical or physical environments.

Our study reveals that MNQ forms a long-lived parent anion with high efficiency at low electron energies. The anion yield exhibits a structured profile at low energies, with prominent signals at 0 eV and around 0.7 eV. The lower lying feature can have contributions from both the (vibrationally excited) π_1_* bound state and from the π_2_* shape resonance, whereas the higher lying feature is assigned to formation of the π_3_* shape resonance. Once the precursor anion state is formed, efficient vibrational relaxation should stabilize the anion in the π_1_* ground state. In turn, DEA pathways leading to the two fragment anions observed (CH_3_^–^ and C_2_H_2_^–^) require electron energies above 4 eV and occur with significantly lower efficiency. In contrast to electron-impact ionization discussed in the Introduction, electron capture is associated with substantially reduced fragmentation.

The confirmation of efficient capture of low-energy electrons by MNQ is particularly relevant in the context of radiation-induced cellular damage. The observed stability of MNQ^–^ suggests that MNQ and possibly its analogues may effectively trap electrons, e.g., secondary, generated by ionizing radiation, facilitating redox cycling and the formation of reactive oxygen species. This behavior indicates its sensitizing function in cancer therapy, where it enhances the cytotoxicity of radiation and chemotherapeutics via oxidative stress and potential DNA damage amplification. These results provide new insights into the fundamental electron-driven processes governing the biological activity of MNQ. The ability of MNQ to stabilize excess electrons at low energies, combined with its inherent redox reactivity, supports its potential role as a redox-active sensitizer. Understanding these mechanisms at the molecular level is essential for the rational design of quinone-based agents in radiotherapy and redox-modulated therapies.

## Data Availability

The data that support the findings of this study are available in a data repository, see https://doi.org/10.5281/zenodo.18662261.
